# The anti-inflammatory effects of *Akkermansia muciniphila* and its derivates in HFD/CCL4-induced murine model of liver injury

**DOI:** 10.1038/s41598-022-06414-1

**Published:** 2022-02-14

**Authors:** Shahrbanoo Keshavarz Azizi Raftar, Fatemeh Ashrafian, Sara Abdollahiyan, Abbas Yadegar, Hamid Reza Moradi, Morteza Masoumi, Farzam Vaziri, Arfa Moshiri, Seyed Davar Siadat, Mohammad Reza Zali

**Affiliations:** 1 Microbiology Research Center (MRC), Pasteur Institute of Iran, Tehran, Iran; 2 Foodborne and Waterborne Diseases Research Center, Research Institute for Gastroenterology and Liver Diseases, Shahid Beheshti University of Medical Sciences, Tehran, Iran; 3 Department of Mycobacteriology and Pulmonary Research, Pasteur Institute of Iran, Tehran, Iran; 4 Clinical Research Departments, Pasteur Institute of Iran, Tehran, Iran; 5 Department of Basic Sciences, School of Veterinary Medicine, Shiraz University, Shiraz, Iran; 6 Gastroenterology and Liver Diseases Research Center, Research Institute for Gastroenterology and Liver Diseases, Shahid Beheshti University of Medical Sciences, Tehran, Iran

**Keywords:** Applied microbiology, Bacteriology

## Abstract

Inflammation plays a critical role in the promotion of hepatocyte damage and liver fibrosis. In recent years the protective role of *Akkermansia muciniphila*, a next-generation beneficial microbe, has been suggested for metabolic and inflammatory disorders. In this study, we aimed to evaluate the effects of live and pasteurized *A. muciniphila* and its extra cellular vesicles (EVs) on inflammatory markers involved in liver fibrosis in a mouse model of a high-fat diet (HFD)/carbon tetrachloride (CCl_4_)-induced liver injury. Firstly, the responses of hepatic stellate cells (HSCs) to live and pasteurized *A. muciniphila* and its EVs were examined in the quiescent and LPS-activated LX-2 cells. Next, the anti-inflammatory effects of different forms of *A. muciniphila* were examined in the mouse model of HFD/CCl_4_-induced liver injury. The gene expression of various inflammatory markers was evaluated in liver, colon, and white adipose tissues. The cytokine secretion in the liver and white adipose tissues was also measured by ELISA. The results showed that administration of live and pasteurized *A. muciniphila* and its EVs leads to amelioration in HSCs activation. Based on data obtained from the histopathological analysis, an improvement in gut health was observed through enhancing the epithelium and mucosal layer thickness and strengthening the intestinal integrity in all treatments. Moreover, live *A. muciniphila* and its EVs had inhibitory effects on liver inflammation and hepatocytes damage. In addition, the tissue cytokine production and inflammatory gene expression levels revealed that live *A. muciniphila* and its EVs had more pronounced anti-inflammatory effects on liver and adipose tissues. Furthermore, EVs had better effects on the modulation of gene expression related to TLRs, PPARs, and immune response in the liver. In conclusion, the present results showed that oral administration of *A. muciniphila* and its derivatives for four weeks could enhance the intestinal integrity and anti-inflammatory responses of the colon, adipose, and liver tissues and subsequently prevent liver injury in HFD/CCL_4_ mice.

## Introduction

Liver disease is the cause of 2–4% of deaths annually worldwide. It encompasses a wide range of clinical conditions, including nonalcoholic fatty liver disease (NAFLD), nonalcoholic steatohepatitis (NASH), alcoholic liver disease (ALD), and viral hepatitis^1,2^. NAFLD is the most common liver disease with a global prevalence of 25%. It is the main cause of advanced liver fibrosis, hepatocellular carcinoma (HCC), and liver failure^3^. A normal liver tissue consists of 5–10% quiescent hepatic stellate cells (HSCs), which are non-parenchymal cells located in the perisinusoidal space of the liver^4^. Activated HSCs are characterized by the expression of fibrosis markers and excessive accumulation of extracellular matrix (ECM) proteins, contributing to the onset and progression of liver fibrosis. Inflammation plays an important role in triggering the activation of HSCs through cytokine secretion by the adjacent Kupffer cells^5^.

A large number of microorganisms inhabit the human gastrointestinal (GI) tract, affecting the host’s health status through maintaining metabolic and immune homeostasis and protecting against pathogens^6^. There is a bilateral connection between the gut and the liver, known as the gut-liver axis; accordingly, the gut microbial products and metabolites are directly conveyed across the portal vein into the liver^7^. Recent evidence shows that changes in the composition of the gut microbiota are associated with many pathological conditions in various liver diseases^8^. Therefore, modulation of the gut microbiota composition can be a potential approach for preventing liver damage^9^.

*Akkermansia muciniphila* (*A. muciniphila*) is an aerotolerant anaerobic mucin-degrading bacterium that inhabits the human GI tract^10,11^. Studies have disclosed the protective and regulatory role of *A. muciniphila* as a next-generation beneficial microbe in the metabolic and immune functions of mice that were fed a high-fat diet (HFD)^12–15^. A series of human and animal studies also support the efficacy of *A. muciniphila* supplementation as a new therapeutic option for the management of obesity and obesity-related metabolic disorders^16,17^. The pasteurized form of *A. muciniphila* can also increase protection against metabolic disorders in HFD-fed mice through increasing the number of goblet cells, which in turn increases the thickness of mucosal layer and improves the intestinal barrier function^14^.

Extracellular vesicles (EVs) are membrane-enclosed vesicles, produced by all types of organisms that play vital roles in intercellular communication and many physiological and pathological processes^18–20^. A variety of medical applications, including vaccine development, adjuvant therapy, delivery systems, and diagnostic and therapeutic applications, have been described for EVs^21^. Besides, EVs derived from the intestinal microbiota can be transmitted to various organs through penetration into the circulatory system and lead to a wide range of immunological responses^22,23^. Evidence suggests that EVs derived from the intestinal microbiota can establish an effective cross-talk between the microbiota and the host immune response, which may be related to the components on the surface of EVs^24^.

In 2019, we found that *A. muciniphila* and its EVs had anti-obesity effects on HFD-fed mice by increasing the mRNA level of peroxisome proliferator-activated receptor (*PPAR*) genes in white adipocytes and inducing immune hemostasis in the colon tissue^13^. Moreover, in 2021, we demonstrated that heat-killed *A. muciniphila* could inhibit HSC activation and induce HSC regression in the lipopolysaccharide (LPS)-activated LX-2 cell line^25^.Accordingly, in the present study, we aimed to evaluate the effects of live and pasteurized *A. muciniphila* and its EVs on inflammatory markers involved in liver fibrosis in a mouse model of HFD/carbon tetrachloride (CCl_4_)-induced liver injury.

In this study, the responses of HSCs to live and pasteurized *A. muciniphila* and its EVs were examined in the quiescent and LPS-activated LX-2 cell line. Next, the anti-inflammatory effects of different forms of *A. muciniphila* and its EVs were examined in the liver tissue of a mouse model of HFD/CCl_4_-induced liver injury, and the gene expression of various inflammatory markers was evaluated in the mouse liver tissue. Moreover, the expression of tight junction (TJ) proteins and inflammatory genes in colon and adipose tissues was examined. To determine if this protection against inflammation occurred in tissues, we also measured the inflammatory and anti-inflammatory cytokine secretion in the liver and white adipose tissues. Finally, the effects of different forms of *A. muciniphila* on the HSC activation and inflammatory markers were compared in vitro and in vivo.

## Materials and methods

### Bacterial culture, pasteurization and EV extraction

*A. muciniphila* MucT strain (ATCC BAA-835) (DSMZ institute, Germany) was cultured in a brain heart infusion (BHI) broth (QUELAB, Canada) supplemented with 0.5% mucin (SIGMA-ALDRICH, St Louis, MO, USA) at 37 °C^10^. After the OD600 reached ~ 1 bacterial suspension was centrifuged at 11,000 g for 20 min, the pellet was washed twice with sterile anaerobic PBS and used for further co-culture experiments and oral gavage in mice. For preparing pasteurized bacteria, a part of the bacterial suspension was also heated at 70 °C for 15 min, as previously described^14^. For viability testing of the pasteurized *A. muciniphila*, the suspension was inoculated on a mucin-based medium and then incubated for at least 1 week at 37 °C in the anaerobic atmosphere. The pasteurized bacteria were aliquot and stored at −80 °C until used. EVs were extracted with ultracentrifugation (200,000 g for 2 h at 4 °C) of the bacterial culture supernatant aforementioned^26^.

### LX-2 activation and treatment

The LX-2 cell line was kindly gifted from Professor Scott L. Friedman (Mount Sinai School of Medicine, New York, NY). LX-2 cells were maintained in complete Dulbecco’s modified Eagle’s medium supplemented with 2 mM of L-glutamine, 100 U/ml of penicillin, 100 μg/ml of streptomycin, and 2% heat-inactivated fetal bovine serum (GIBCO-INVITROGEN, Carlsbad, CA). For LX-2 activation, the lipopolysaccharides (LPS) from *Escherichia coli* 0111:B4 (SIGMA-ALDRICH) was used at concentration of 0.01 µg/ml for 6 h as previously described^25^. Afterwards, unstimulated LX-2 and LPS-activated cells were inoculated with live (Lam) and pasteurized (Pam) *A. muciniphila* at different multiplicities of infection (MOIs 1, 10, 100), and EVs of varying concentrations (1, 10, 50 µg/ml) for 24 h in the CO_2_ incubator at 37 °C. Each experiment was performed in duplicate, and repeated at least three times.

### Total RNA extraction and qRT-PCR analysis

Total RNA was extracted from LX-2 cells using RNeasy Plus Mini Kit (QIAGEN, Germany) following the supplier’s protocol. The quantity and quality of RNA was verified via agarose gel electrophoresis and NanoDrop spectrophotometer (ND-1000, THERMO SCIENTIFIC, USA). The purified RNA was converted to cDNA using the BIOFACT RT-Kit (BIOFACT, South Korea) according to the manufacturer’s protocol. The qRT-PCR amplification was done on a ROTOR-GENE Q (QIAGEN, Germany) real-time PCR system by using BIOFACT 2X Real-Time PCR Master Mix (BIOFACT, South Korea). The GAPDH served as the reference gene. The oligonucleotide sequences used in this work are presented in Table 1.

**Table 1:** Oligonucleotide primers used in real-time PCR in cell line and mice tissues.

Target gene	Primer designation	Oligonucleotide sequence (5′–3′)	Product size (bp)
m-*rpl*-19	Rpl-F Rpl-R	TCAGCCACAACATTCTCA GCACCTCCAACAGTAAGT	138
m-*tlr*-5	Tlr5-F Tlr5-R	AAGACTGCGATGAAGAGGAA GGTGATGACGAGGAATAGAGT	92
m-*tlr*-9	Tlr9-F Tlr9-R	TCAGCCACAACATTCTCA GCACCTCCAACAGTAAGT	138
m-*il*-1β	IL-1b-F IL-1b-R	AACAACTACTCAGAAACACAAG GCAGAACTCAGGAATGGA	130
m-*il*-6	IL6-F IL6-R	TCCATCCAGTTGCCTTCT TAAGCCTCCGACTTGTGAA	137
m-*il*-10	IL10-F IL10-R	GCACTACCAAAGCCACAAG AGTAAGAGCAGGCAGCATAG	85
m-*igf*	Igf-F Igf-R	TCGTGGGATGGGTGCTTT TGAAGACAGTAGGGAAGAGACAAG	154
m-*ppar*-α	PPARa-F PPARa-R	CACTTGCTCACTACTGTCCTT GATGCTGGTATCGGCTCAA	110
m- *ppar*-γ	PPARg-F PPARg-R	GGTGCTCCAGAAGATGACAGA TCAGCGGGTGGGACTTTC	154
m-*ocldn*	Ocldn-F Ocldn-R	TTGAAAGTCCACCTCCTTACAGA CCGGATAAAAAGAGTACGCTGG	129
m-*cldn*-1	Cldn1-F Cldn1-R	TCTGCCACTTCTCACTTCCA GCCTATACCCTTGCTCTCTGT	95
m-*cldn*-2	Cldn2-F Cldn2-R	TGGAAGATGGTGATGGGATT CCCTTGGAAAAGCCAACCG	128
*gapdh*	GAPDH-F GAPDH-R	TCAACGGATTTGGTCGTATTG TGGAAGATGGTGATGGGATT	211
*ppar*-α	PPARa-F PPARa-R	GAGCGTTGTCTGGAGGTT GAAGTGGTGGCTAAGTTGTTG	115
*ppar*-γ	PPARg-F PPARg-R	TACGAAGACATTCCATTCACAAGA CTCCACAGACACGACATTCA	199
*tlr*-5	Tlr5-F Tlr5-R	TCCCTGAACTCACGAGTCTTT GGTTGTCAAGTCCGTAAAATGC	109
*tlr*-9	Tlr9-F Tlr9-R	CTGCCACATGACCATCGAG GGACAGGGATATGAGGGATTTGG	121

### Animal experiments

All animal studies were carried out in compliance with the ARRIVE guidelines. Twenty-five male wild-type C57BL/6 mice were obtained at the age of 8 weeks from Pasteur Institute of Iran (Tehran Iran). Acclimatization was performed in 12 h light, 22–23 °C, and 40% humidity condition with a free access to standard diet (SD) (Standard Rodent Diet A03; SAFE, Augy, France) and autoclaved water. Then, mice were individually housed in autoclaved cages and sterile hardwood chip bedding during the experiment. This study followed the institutional guidelines regarding the care and use of laboratory animals. The study protocol was approved by the Animal Experiment Committee Pasteur Institute of Iran (IR.PII.REC.1399.029).

**Figure 1:**
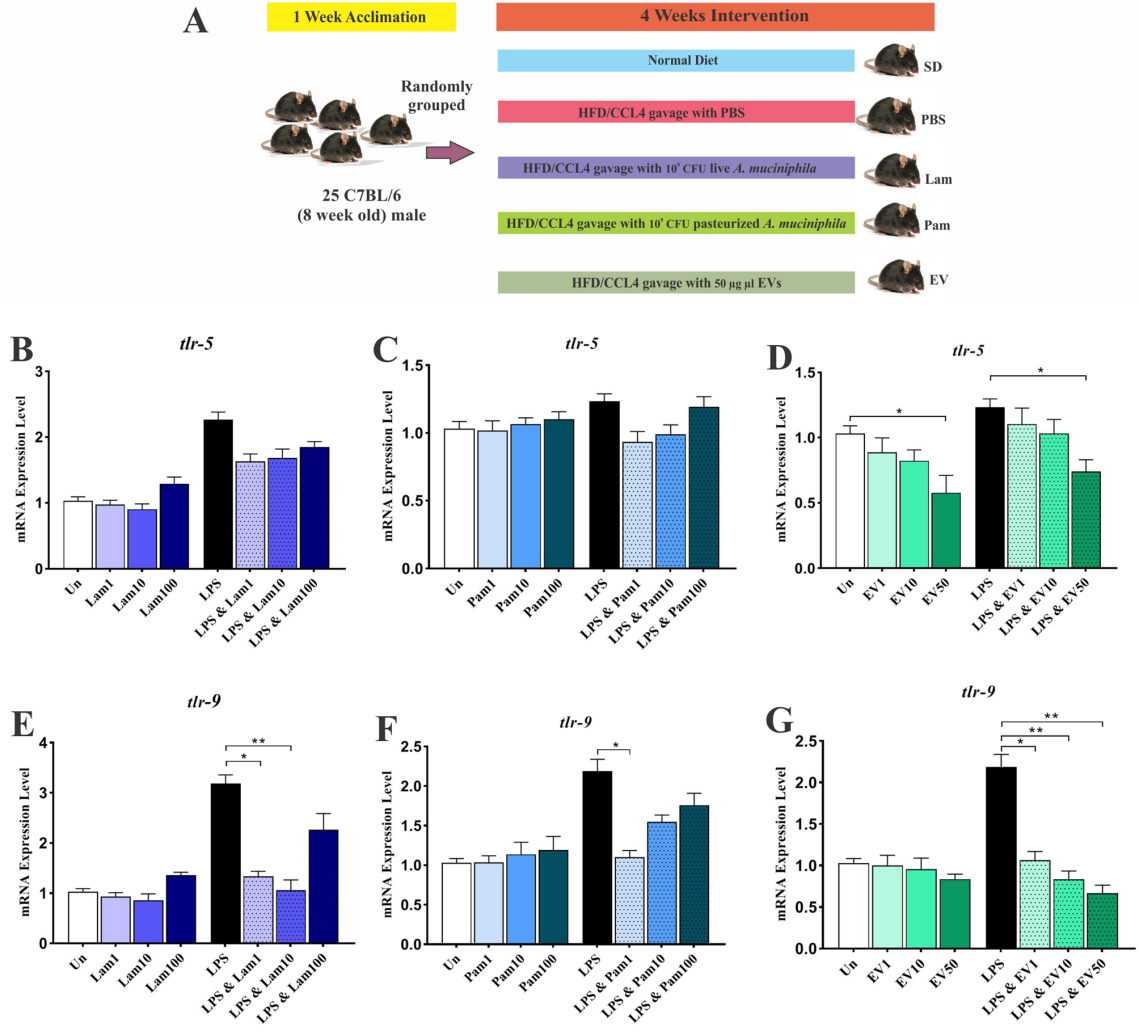
(**A**) Study design of the animal experiment. Anti-inflammatory effects of all *A. muciniphila* supplementations in LX-2 cell line. The mRNA Level of *tlr*-5 in quiescence and LPS-activated LX-2 cells treated with (**B**) Lam (**C**) Pam and (**D**) EVs; and *tlr*-9 in quiescence and LPS-activated LX-2 cells treated with (**E**) Lam (**F**) Pam and (**G**) EVs. Un: untreated cells, Lam: live *A. muciniphila*, Pam: pasteurized *A. muciniphila*, EV: extra cellular vesicles of *A. muciniphila*. Data are expressed as mean ± SD (*n* = 5). *p < 0.05, **p < 0.01 and ***p < 0.001 by post hoc Turkey’s one-way ANOVA statistical analysis.

For liver injury induction, CCl_4_ accompanied by a high-fat diet (260 HF 60% energy from butter, safe diet, France) was used. The HFD animals were intraperitoneally injected with 2 ml/kg body weight of 10% CCl4 solution in olive oil (SIGMA-ALDRICH, St. Louis, MO, USA) twice a week for 4 weeks^27^. Mice were randomly grouped (*n* = 5), healthy control animals received the SD without intervention, vehicle (HFD/CCL4 + 200 µl sterile PBS), Lam (HFD/CCL_4_ + 10^9^ CFU/200 µl live *A. muciniphila*), Pam (HFD/CCL_4_ + 10^9^ CFU/200 µl pasteurized *A. muciniphila*) and EV (HFD/CCL_4_ + 50 µg protein/200 µl) were given by daily oral gavage for 4 weeks (Fig. 1 A). At the end of the treatments, the mice were sacrificed by cervical dislocation and liver, colon and white adipose tissues were snap frozen with liquid nitrogen, and stored at −80 °C. The liver and colon tissues were further used for histopathological analysis and blood samples were taken without prior food fasting, serum was collected and stored at –80 °C until further analysis.

### Histopathological evaluation and histometric analysis

Liver and colon specimens were immediately fixed in 10% neutral buffered formalin. Paraffin-embedded liver and colon tissues were stained with hematoxylin and eosin (H&E) method^28^. The thickness of the epithelium and mucosal layer were then evaluated by an expert pathologist, blind to the study groups. Histological pictures of colon and liver were taken using a light microscope (OLYMPUS SX-21) equipped with a digital camera (TRUECHROME II).

For histometrical study, Dino Lite digital lens and Dino Capture 2 Software and light microscope were used. Furthermore, histometrical structures of colon were analyzed, including thickness of mucosal layer and the epithelium. A microscopic histologic injury score was assigned to each mouse. Scoring was based on infiltration of inflammatory cells. Each category was scored with a score of 0 indicating no infiltration of inflammatory cells and a score of 3 indicating infiltration of inflammatory cells^29,30^. Number of bi-nucleated hepatocytes per mm2 was calculated in 20 randomly selected fields of each section using Dino Lite digital lens and Dino Capture 2 Software and light microscope^31^.

### Measurement of serum aminotransferases and liver cytokine

Serum level of alanine transaminase (ALT) and aspartate transaminase were assessed to show the degree of liver injury using commercial assay kits according to the instructions of manufacturers. The level of IL-10, TNF-α and IL-6 in liver and adipose tissues were determined by using ZELLBIO GmbH ELISA kit (Germany) according to the manufacturer’s instructions, and analyzed with Bio-Plex Manager 6.1 software (BIO-RAD, USA). This cytokine assays were performed in duplicate.

### Tissue RNA extraction and qRT-PCR analysis

Frozen liver, colon and white adipose tissues were homogenized in 1 ml of TRIZOL (33 BS410, Bio Basic, Canada) using a Precellys 24homogenizer and the total RNA was extracted according to the manufacturer’s instructions. The genomic DNA was removed using DNase I (QIAGEN), cDNA was synthesized and qRT-PCR amplification was carried out as mentioned earlier. The relative expression of target genes was assessed using the comparative cycle threshold (Ct) method. The *rpl*-19 served as the normalizer genes.

### Statistical analysis

GRAPHPAD PRISM 8.0 (GRAPHPAD Software Inc, CA, United States) was exploited for calculating changes in gene expression and cytokine production. Differences between groups were calculated using one-way analysis of variance (ANOVA) followed by Tukey’s post hoc test (for multiple comparisons between more than two groups). Results are shown as mean ± SD (standard deviation) of the mean of at least three experiments. A *p* value less than 0.05 were considered statistically significant.

### Approval for animal experiments

The study was approved by the Animal Experiment Committee of Pasteur Institute of Iran (IR.PII.REC.1399.029), and the Institutional Ethical Review Committee of Research Institute for Gastroenterology and Liver Diseases at Shahid Beheshti University of Medical Sciences (Project No. IR.SBMU.RIGLD.REC.1395.211).

## Results

### *A. muciniphila *attenuates mRNA level of *tlr*-5 and *tlr*-9 in LPS-activated LX-2 cells

To evaluate the effects of *A. muciniphila* and its derivatives on mRNA levels of *tlr*-5 and *tlr*-9 genes, different MOIs (1, 10, and 100) and concentration of EVs (1, 10, and 50 µg) were used in quiescent and LPS-activated LX-2 cells. As shown in Fig. 1B–G, in quiescent LX-2 cells, none of the live and pasteurized treatments altered the expression of tlr-5 and *tlr*-9 genes, while EVs in a concentration of 50 µg had an inhibitory effect on the expression of *tlr*-5 in comparison with untreated control cells (*p* value 0.03). In LPS-stimulated LX-2 cells also, just EV in concentrations of 50 µg could significantly reduce *tlr*-5 gene mRNA level (*p* value 0.012). In LPS-activated LX-2 cells, all concentrations of EV were able to down-regulate the *tlr*-9 gene significantly (*p* value 0.04, 0.003 and 0.001 for 1, 10, and 50 µg respectively), while Lam at MOI 1 (*p* value 0.05) and 10 (*p* value 0.02) and Pam at MOI1 (*p* value 0.05) could reduce the expression of *tlr*-9 gene. Therefore, although all treatments had an inhibitory effect on the mRNA level of the *tlr*-5 and *tlr*-9 genes in the activated LX-2 cells, the EVs effects were more pronounced and almost no significant effect was observed on the quiescent cells.

### *A. muciniphila* significantly up-regulates PPARs gene expression in LX-2 cells

**Figure 2:**
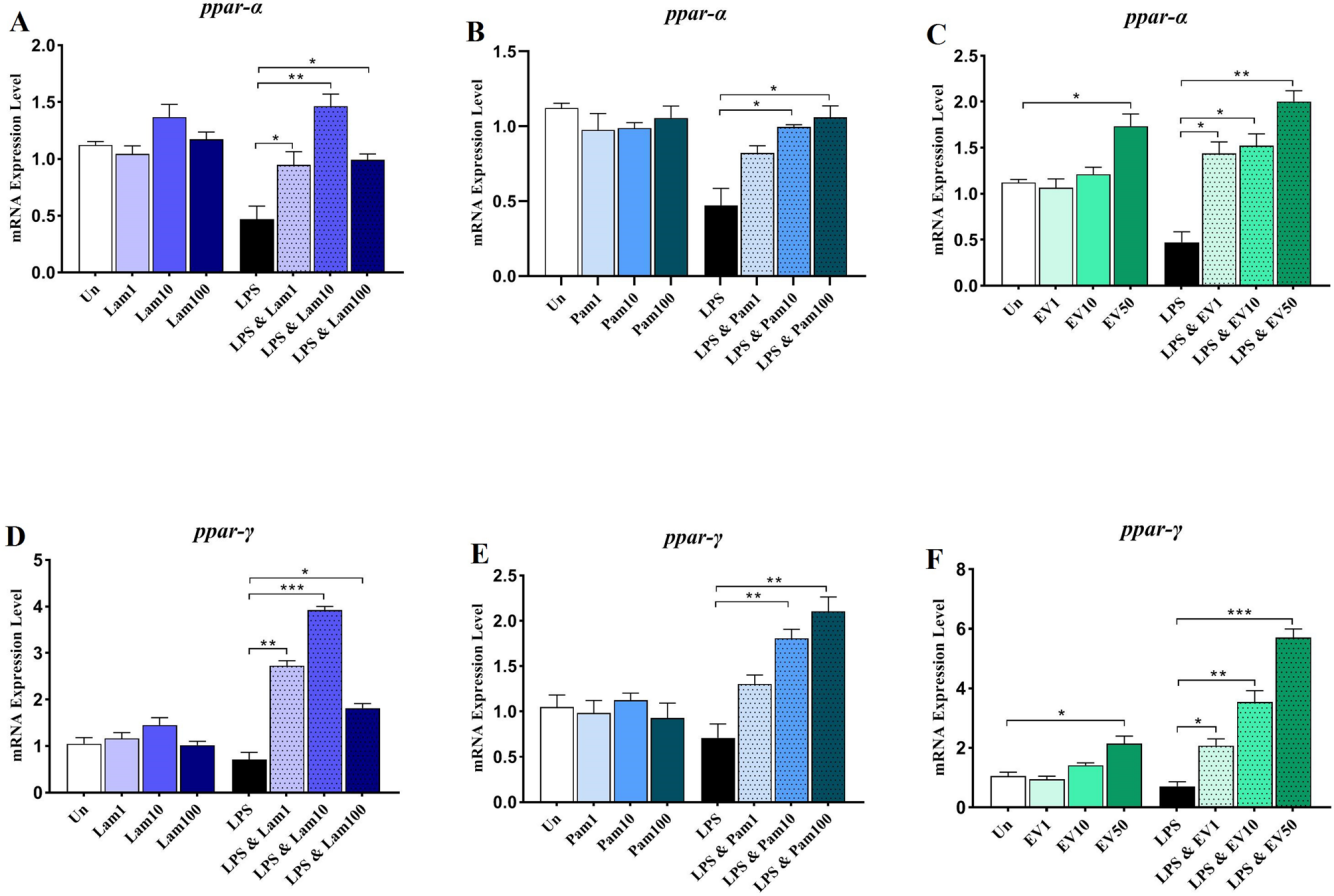
Anti-inflammatory effects of all *A. muciniphila* supplementations in LX-2 cell line. The mRNA Level of *ppar-α* in quiescence and LPS-activated LX-2 cells treated with (**A**) Lam (**B**) Pam and (**C**) EVs; and *ppar-γ* in quiescence and LPS-activated LX-2 cells treated with (**D**) Lam (**E**) Pam and (**F**) EVs. Un: untreated cells, Lam: live *A. muciniphila*, Pam: pasteurized *A. muciniphila*, EV: extra cellular vesicles of *A. muciniphila*. Data are expressed as mean ± SD (*n* = 5). *p < 0.05, **p < 0.01 and ***p < 0.001 by post hoc Turkey’s one-way ANOVA statistical analysis.

The effect of live and pasteurized *A. muciniphila* and its EVs on gene expression of anti-inflammatory factor *ppar-α* and *ppar-γ* was also investigated in quiescent and LPS-stimulated LX-2 cells. As shown in Fig. 2 in quiescent cells, treatment with different live and pasteurized bacterial MOIs and EV concentrations did not significantly alter the expression of the *ppar-α* and *ppar-γ* gene, whereas EV in concentrations of 50 µg was able to significantly increase the mRNA level of *ppar-α* and *ppar-γ* (*p* value 0.01 and 0.03 respectively).

In LPS-activated cells, a significant mRNA level of *ppar-α* gene was induced compared to untreated control by different treatments. Among them, Lam and EV at all three MOI and concentrations and Pam at MOI10 and 100 showed a significant effect on induction of *ppar-α* gene (Fig. 2A–C). However, in LPS-activated LX-2 cells, a significant mRNA level of *ppar-γ* was induced by different treatments compared to untreated control cells, among which bacterial MOI10 and EV 50 showed a significant effect on induction of *ppar-γ* gene (*p* value < 0.0001 and < 0.0001 respectively) (Fig. 2D–F). Thus, almost all treatments were able to induce the expression of *ppar-α* and *ppar-γ* genes, although the best effect was observed in EV treatments in a dose-dependent manner.

### *A. muciniphila* and its derivatives could maintain intestinal homeostasis by modulating inflammation

**Figure 3:**
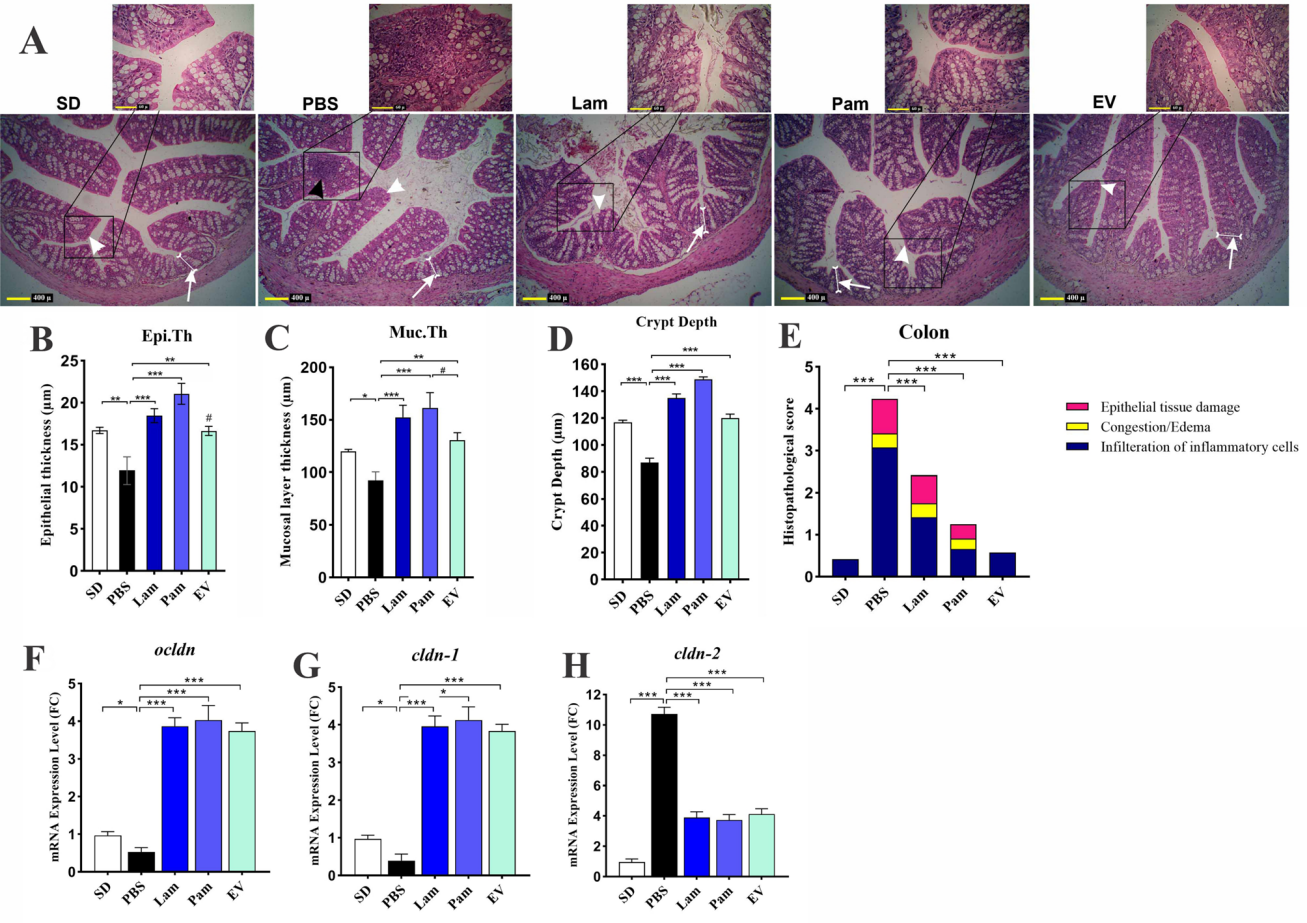
(**A**) Histological structure of colon in different study groups using H&E staining. The infiltration of inflammatory cells (black arrow) was observed in the mucous membrane of the PBS group. The decrease in thickness of mucosal layer (white arrows) and thickness of the epithelium (white arrowheads) of the colon were seen in histological sections of PBS group. An increase in thickness of the epithelium (white arrowheads) and thickness of mucosal layer (white arrows) was seen in Pam, Lam, and EV groups, compared to that in PBS group. histometric analysis shows the difference of (**B**) epithelium thickness (Epi.Th) and (**C**) the mucosal layer thickness (Muc.Th) (**D**) crypt depth and (**E**) histopathological score among the study groups. Assessment of a live and pasteurized *A. muciniphila* and its EVs effects on mRNA expression of tight junction proteins gene in the colon tissue. (**F**) ocldn, (**G**) cldn-1 and (H) cldn-2. SD: standard diet, PBS: HFD/CCL4 + PBS, Lam: HFD/CCL4 + *A. muciniphila* (109 CFU), Pam: HFD/CCL4 + pasteurized *A. muciniphila* (109 CFU) and EV: HFD/CCL4 + 50 µg EVs. Data are expressed as mean ± SD (*n* = 5). * *p* < 0.05, ** *p* < 0.01, *** *p* < 0.001 in comparison with PBS group and ## *p* < 0.01 in comparison with Pam by post hoc Turkey’s one-way ANOVA statistical analysis.

As shown in Fig. 3A, the PBS group revealed the infiltration of inflammatory cells (Grade 2) in the mucous membrane of the colon. The thickness of epithelium showed a significant decrease in PBS group (11.92 ± 1.64 μm), compared to that in SD group (16.7 ± 0.37 μm) (*p* value < 0.05) (Fig. 3B). The thickness of mucosal layer showed a significant decrease in PBS group (92.58 ± 7.8 μm), compared to that in SD group (119.95 ± 1.97 μm) (*p* value < 0.05). The maximum thickness of mucosal layer (161.45 ± 14.5 μm) and epithelium (21.05 ± 1.25 μm) was shown in Pam group (Fig. 3C). Furthermore, the thickness of mucosal layer and epithelium showed a significant increase in Lam and EV, compared to that in PBS group (*p* value < 0.05). As demonstrated in Fig. 3D, Crypt depth showed a significant increase in EV (119.9 ± 3.1 µm), Pam (148.9 ± 1.8 µm) and Lam (135.1 ± 3.2 µm) groups, compared to that in PBS group (*p* value < 0.000). Furthermore, crypt depth showed a significant increase in Pam and Lam groups, compared to that in SD group (116.8 ± 1.6 µm) (*p* value < 0.05). Moreover, the crypt depth in the colon showed a significant decrease in PBS group (86.9 ± 1.6 µm), compared to that in all groups (*p* value < 0.000).

By using a histopathological score system, as described previously^30^ histologic injury score of the mice colon tissue was categorized according to the following characterizes: epithelial tissue damage, infiltration of inflammatory cells, hemorrhagic congestion and edema of the mucosa in the three categories range from 0 (no injury) to 3 (severe injury). The overall score was the sum of each component score (Fig. 3E). The total score of each group (Mean ± SE) was as follows: SD group, 0.42 ± 0.22; PBS group, 4.25 ± 0.14; Lam group, 2.42 ± 0.22; Pam group, 1.25 ± 0.14 and EV group 0.58 ± 0.08. Statistical analysis of the histopathological score showed the following: PBS vs SD with *p* value < 0.000; Lam vs PBS with *p* value < 0.000; Pam vs PBS with *p* value < 0.000; and EV vs PBS with *p* value < 0.000.

These observations were accompanied by a significant increase in mRNA level of tight junction proteins *ocldn* and *cldn*-1 and a decrease in *cldn*-2 in the colon of mice treated with live and pasteurized *A. muciniphila* and its EVs (Fig. 3 F–H). Although there was a significant difference in the thickness of mucosal layer between treatments, their ability to induce tight junction protein genes was the same.

### Live *A. muciniphila* and its EVs had anti-inflammatory effects on liver tissue of HFD/CCL4-treated mice

**Figure 4:**
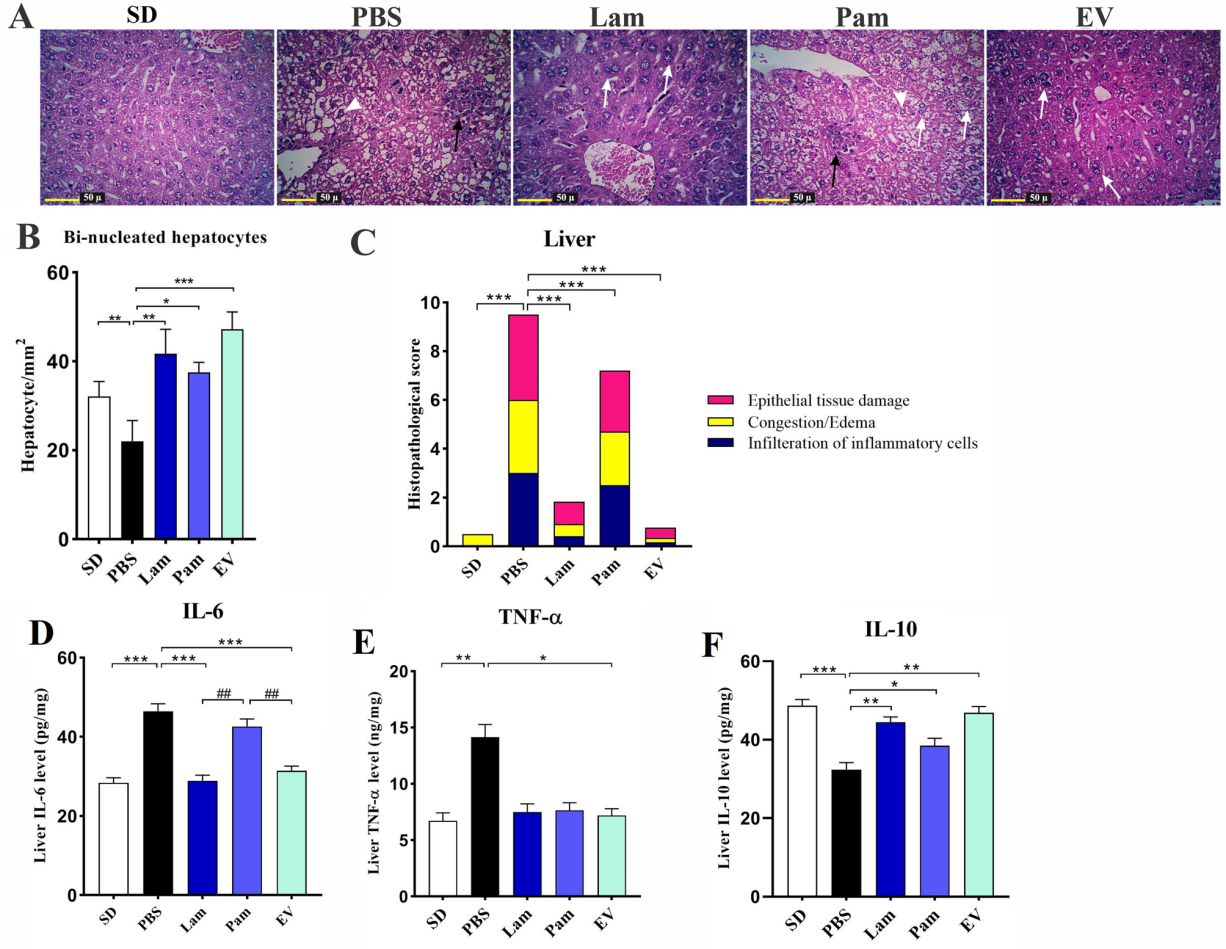
(**A**) The effect of a live and pasteurized *A. muciniphila* administration on the histopathological structure of the liver in different study groups (H&E). In the PBS group, infiltration of inflammatory cells and spotty necrosis of hepatocytes (Black arrows), lipid micro and macro vesicles (White arrowheads) were presented in the cytoplasm of hepatocytes. Lam and EV groups: without histopathological changes and the presence of bi-nucleated cells (white arrows). Pam group: infiltration of inflammatory cells and spotty necrosis of hepatocytes (Black arrows), lipid micro and macro vesicles (White arrowheads) and the presence of bi-nucleated cells (white arrows). The number of bi-nucleated hepatocytes in histological structure of liver in different study groups (mean ± SD). (**B**) Counting of bi-nucleated hepatocytes per mm2 (**C**) histopathological score. The liver tissue cytokines level (**D**) IL-6, (E) TNF-α and (F) IL-10. SD: standard diet, PBS: HFD/CCL4 + PBS, Lam: HFD/CCL4 + *A. muciniphila* (109 CFU), Pam: HFD/CCL4 + pasteurized *A. muciniphila* (109 CFU) and EV: HFD/CCL4 + 50 µg EVs. Data are expressed as mean ± SD (*n* = 5). * *p* < 0.05, ** *p* < 0.01, *** *p* < 0.001 in comparison with PBS group and ## *p* < 0.01 in comparison with Pam by post hoc Turkey’s one-way ANOVA statistical analysis.

Liver histopathological analysis confirmed that acute liver injury was established in PBS group comparing to SD group (Fig. 4A). In H&E staining, no histopathological changes were observed in SD group, while infiltration of inflammatory cells in the form of spotty necrosis and necrosis of hepatocytes along with the lipid micro and macro vesicles in the cytoplasm of hepatocytes was observed in PBS group. As shown in Fig. 4B, the number of bi-nucleated hepatocytes showed a significant decrease in PBS group, compared to that in SD group (*p* value < 0.05). Furthermore, number of bi-nucleated hepatocytes showed a significant increase in EV, Lam and Pam groups, compared to that in SD as well as PBS groups (*p* value < 0.05 and < 0.05, respectively). The presence of bi-nucleated hepatocyte cells indicated hepatocyte regeneration without any other histopathological changes. In Pam group, although infiltration of inflammatory cells, spotty necrosis, necrosis of hepatocytes, and lipid micro and macro vesicles was observed in the cytoplasm of hepatocytes, the presence of bi-nucleated hepatocyte cells indicating hepatocyte regeneration was also observed. In addition, by using a histopathological score system, as described previously^32,33^ histologic injury score of the liver tissue was categorized according to the following characterizes: necrosis, infiltration of inflammatory cells, steatosis in the four categories range from 0 (no) to 4 (severe). The overall score was the sum of each component score (Fig. 4C). The total score of each group (Mean ± SE) was as follows: SD group, 0.5 ± 0.14; PBS group, 9.5 ± 0.28; Lam group, 1.83 ± 0.16; Pam group, 6.83 ± 0.44 and EV group 0.75 ± 0.13. Statistical analysis of the histopathological score showed the following: PBS vs SD with *p* value < 0.000; Lam vs PBS with *p* value < 0.000; Pam vs PBS with p value < 0.000; and EV vs PBS with *p* value < 0.000. In addition, there were no significant differences observed between EV vs SD.

To investigate the anti-inflammatory effects of live and pasteurized *A. muciniphila* and its EVs, the level of TNF-α, IL-6, and IL-10 in mice liver tissue was also assessed. As shown in Fig. 4D–F, asignificant increase in the level of IL-6 (*p* value 0.0006), TNF-α (*p* value 0.001) and decrease in IL-10 (*p* value 0.0002) was observed in PBS group compared to SD group indicating inflammation was induced in the liver tissue of HFD/CCL_4_ treated mice. Although oral gavage with live *A. muciniphila* could decrease TNF-α, this effect was not statistically significant (*p* value 0.1), while administration of EVs could remarkably decrease the level of TNF-α (*p* value 0.01). Moreover, oral gavage with live *A. muciniphila* and its EVs could significantly decrease the level of IL-6 (*p* value 0.001), while pasteurized form could not significantly change in IL-6 level compared to PBS group. The effect of pasteurized *A. muciniphila* also was not significant on TNF-α cytokines, while it significantly increased the tissue level of IL-10(*p* value 0.03). Although oral gavage with all treatments could decrease the level of IL-6 and TNF-α and increase IL-10, these anti-inflammatory effects were remarkable in Lam and EV groups.

Moreover, assessment of serum aminotransferases level showed a significant increase in ALT and AST level of the PBS in comparison with the SD group (*p* value < 0.0001), indicating liver injury was established in PBS group. As shown in Table 2, oral gavage with Lam, Pam, and EVs was able to decrease the level of ALT, AST in study groups.

**Table 2:** The effect of a live and pasteurized *A. muciniphila* and its EVs administration on serum level of aminotransferases after 4 weeks (*n* = 5 for each group).

Variables	Study groups (mean ± SD)					*P* value			
	SD	PBS	Lam	Pam	EV	SD vs. PBS	Lam vs. PBS	Pam vs. PBS	EV vs. PBS
ALT (U/dl)	71.00 ± 6.43	162.00 ± 6.24	85.18 ± 9.08	121.3 ± 5.84	84.18 ± 6.67	** < 0.0001**	** < 0.0001**	**0.002**	** < 0.0001**
AST (U/dl)	86.74 ± 3.88	181.9 ± 5.49	83.82 ± 6.12	92.26 ± 5.23	85.16 ± 4.86	** < 0.0001**	** < 0.0001**	** < 0.0001**	** < 0.0001**

SD; standard-diet, Lam; live *A. muciniphila*, Pam; pasteurized *A. muciniphila*, EV; extracellular vesicle, ALT; alanine aminotransferase, AST; aspartate aminotransferase, Bold *p* value are indicated statically significant.

### *A. muciniphila* demonstrates inhibitory effect on inflammatory genes in liver tissue of HFD/CCL4-treated mice

In the present study, the anti-inflammatory effect of live and pasteurized *A. muciniphila* and its EVs was investigated by inhibiting the expression of TLRs and inflammatory cytokines such as *il-1β*, *il-6,* and anti-inflammatory *il-10* in the liver tissue of the HFD/CCL_4_ murine model.

**Figure 5:**
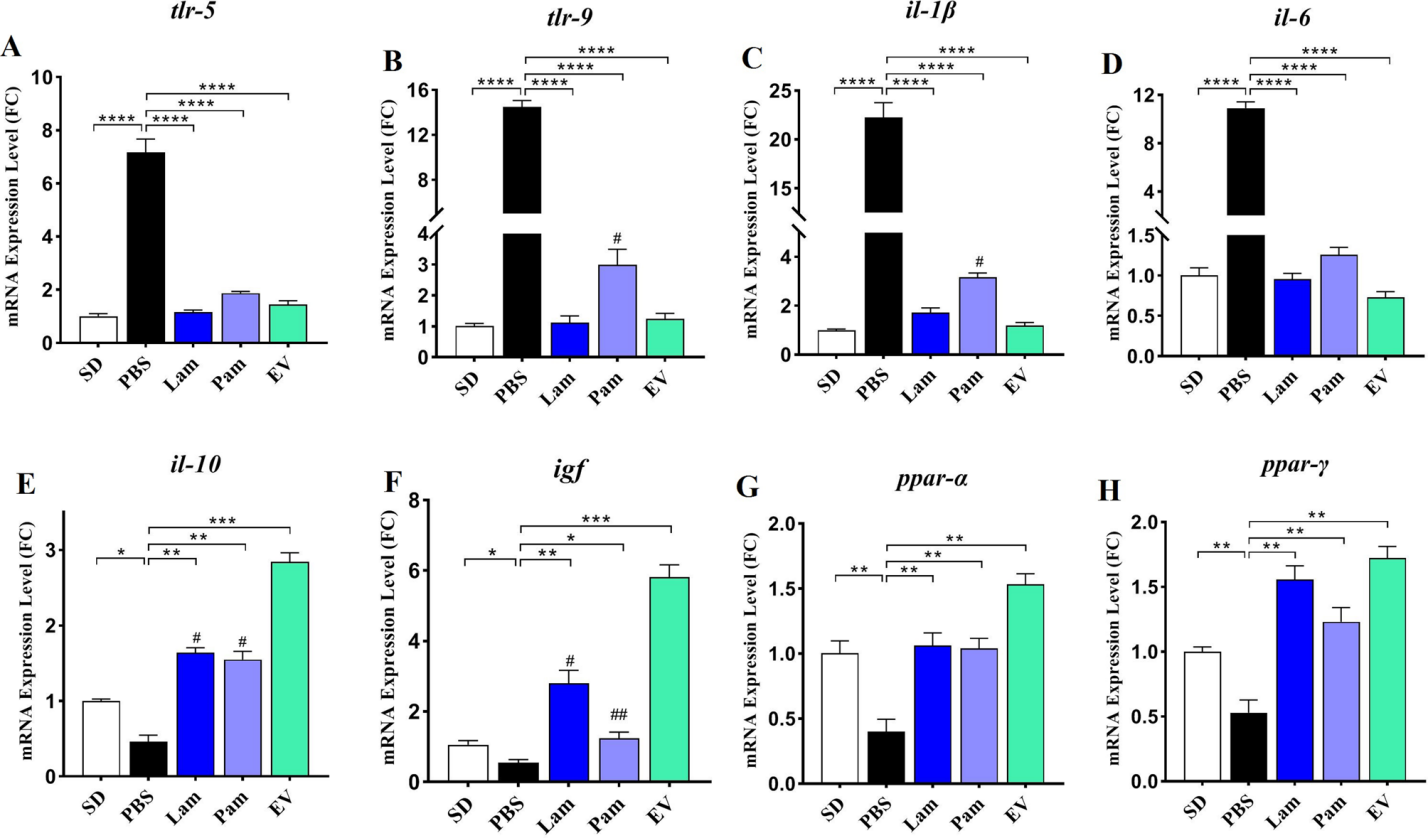
Hepatic mRNA expression of inflammatory-related genes, (**A**) *tlr*-5; (**B**) *tlr*-9; (**C**) *il*-1β; (**D**) *il*-6; and anti-inflammatory-related genes (**E**) *il*-10; (F) *igf*; (**G**) *ppar*-α and (H) *ppar*-γ. SD: standard diet, PBS: HFD/CCL4 + PBS, Lam: HFD/CCL4 + A. muciniphila (10^9^ CFU), Pam: HFD/CCL4 + pasteurized A. muciniphila (10^9^ CFU) and EV: HFD/CCL4 + 50 µg EVs. Data are expressed as mean ± SD (*n* = 5). * *p* < 0.05, ** *p* < 0.01 and *** *p* < 0.001 in comparison with PBS group, # *p* < 0.05 and ## *p* < 0.01in comparison with EV by post hoc Turkey’s one-way ANOVA statistical analysis.

As shown in Fig. 5 A-E, HFD/CCL_4_ remarkably elevated *tlr*-5 and *tlr-*9 mRNA levels which in turn led to an increase in inflammatory cytokines and a decrease in *il-10* in the PBS group (*p* value < 0.0001). Administration of Lam, Pam, and EVs were able to significantly decrease the expression of *tlr*-5 and *tlr*-9 genes compared to the PBS group (*p* value < 0.0001, < 0.0001 and < 0.0001, respectively). This bacterium and its derivatives also regulated the inflammatory and anti-inflammatory cytokines by reducing the expression of *il-1β* and *IL-6* and increasing the *il-10* consequently induced immune homeostasis in liver tissue. Interestingly, EV showed the highest effect on *il-10* induction and *il-1β* inhibition between the groups.

### *A. muciniphila* shows anti-inflammatory effects on liver tissue of HFD/CCL4-treated mice through up-regulating of PPARs gene expression

The effect of live and pasteurized *A. muciniphila* and its EVs was investigated on gene expression of anti-inflammatory factors *ppar-α*, *ppar-γ* and *igf* in liver tissue. As shown in Fig. 5 F–H, *ppar-α*, *ppar-γ,* and *igf* expression were significantly reduced in the PBS group which hepatic injury was induced by HFD/CCL_4_. Oral gavage with all treatments was able to considerably induced the mRNA level of these anti-inflammatory factors *ppar-α*, *ppar-γ,* and *igf* in liver tissue of study groups in comparison with PBS group, whereas the effect of EVs was more noticeable than live and pasteurized *A. muciniphila* especially in case of *igf* and *ppar-α* (*p* value < 0.0001 and < 0.0001).

### EVs had a remarkable anti-inflammatory effect on white adipose tissue of HFD/CCL_4_-treated mice

**Figure 6:**
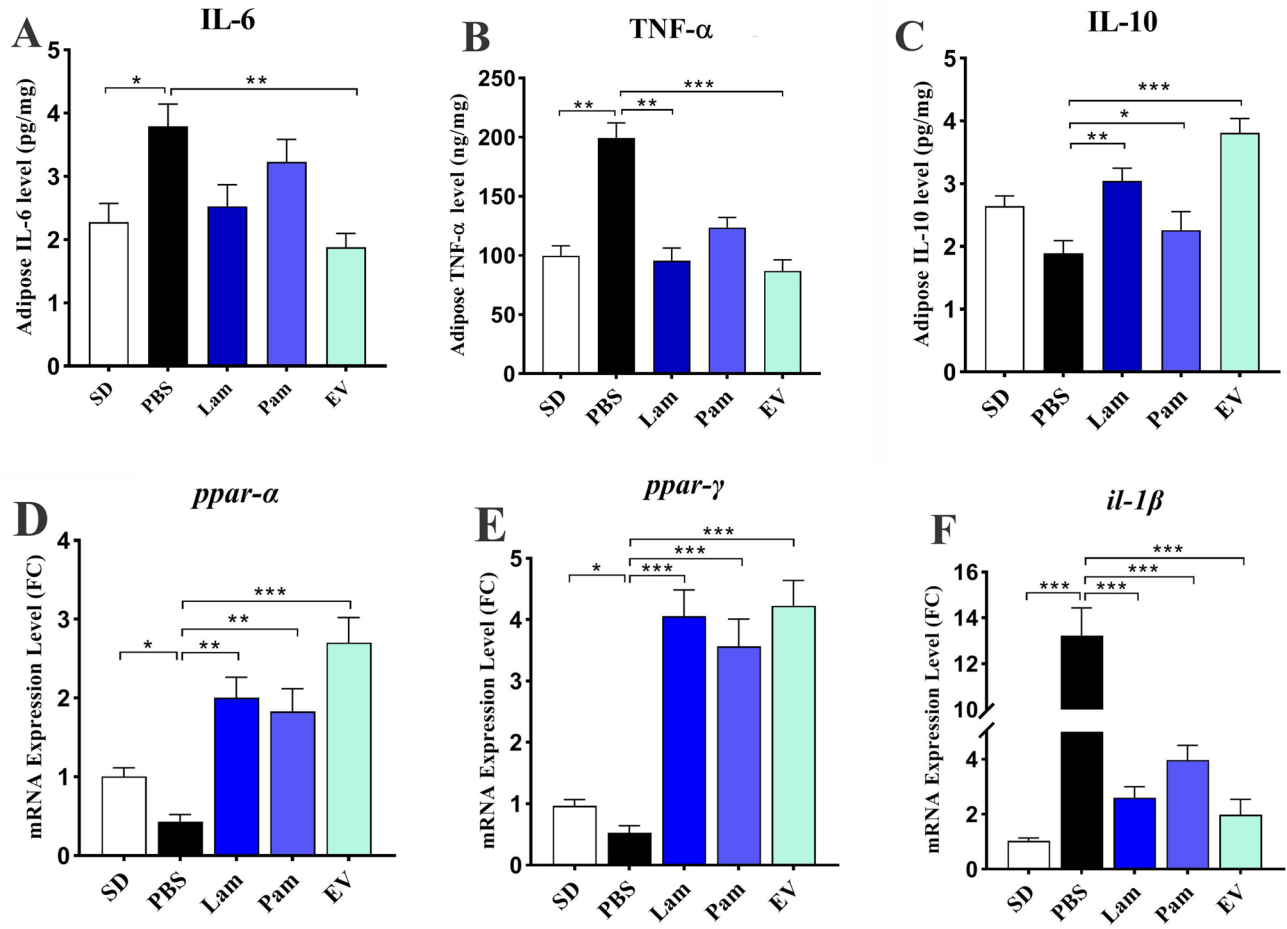
The adipose tissue cytokines level (**A**) IL-6; (**B**) TNF-α and (**C**) IL-10. Adipose tissue mRNA level of (**G**) *ppar*-α, (**H**) *ppar*-γ and (**C**) *il*-1β. SD: standard diet, PBS: HFD/CCL4 + PBS, Lam: HFD/CCL4 + *A. muciniphila* (10^9^ CFU), Pam: HFD/CCL4 + pasteurized *A. muciniphila* (10^9^ CFU) and EV: HFD/CCL4 + 50 µg EVs. Data are expressed as mean ± SD (*n* = 5). * *p* < 0.05, ** *p* < 0.01 and *** *p* < 0.001 by post hoc Turkey’s one-way ANOVA statistical analysis.

To investigate the anti-inflammatory effects of live and pasteurized *A. muciniphila* and its EVs, the level of TNF-α, IL-6, and IL-10 in mice adipose tissue was assessed. As shown in Fig. 6 A-C, a significant increase in level of IL-6 (*p* value 0.01) and TNF*-α* (*p* value 0.02) was observed in PBS group compared to SD group indicating inflammation was induced in adipose tissue. Although oral gavage with live and pasteurized *A. muciniphila* could decrease IL-6, this effect was not statistically significant, while administration of EVs could remarkably decrease the level of IL-6 (*p* value 0.0003). Oral gavage with live *A. muciniphila* and its EVs were also decreased TNF-α (*p* value 0.008 and 0.0004 respectively) and increased IL-10 (*p* value 0.009 and 0.0001 respectively). The effect of pasteurized *A. muciniphila* on TNF-α and IL-6 was not statically significant although IL-10 was significantly changed (*p* value 0.04). Although oral gavage with all treatments could decrease the level of IL-6 and TNF-α and increase IL-10, these anti-inflammatory effects were remarkable in Lam and EV groups.

The anti-inflammatory effects of *A. muciniphila* and its EVs were accompanied by an increase in the mRNA level of *ppar-α* and *ppar-γ* along with decrease in *il-1β* in mice white adipose tissue. Although live and pasteurized *A. muciniphila* was able to enhance the expression of *ppar-α*, the effect of EVs was more noticeable (*p* value < 0.0001). All treatments had significant increasing effect on *ppar-γ* and decreasing *il-1β* in adipose tissue, while administration of HFD/CCL_4_ remarkably down-regulated *ppar-α* and *ppar-γ* and up-regulated *il-1β* in PBS group (Fig. 6 D-F).

## Discussion

Liver inflammation is considered the main cause of liver disease and hepatic tissue damage, leading to the progression of liver fibrosis and eventually HCC^34^. The main pathological mechanism underlying systemic inflammation is disruption of the intestinal epithelial integrity due to a decrease in TJ proteins. Liver is the first organ to be affected by this disturbance, as seen in the pathophysiology of different liver diseases^35,36^. The results of this study showed that four weeks of oral gavage with live and pasteurized *A. muciniphila* and its EVs could successfully maintain homeostasis in the colon tissue and increase mucosal thickness and crypt depth. This improvement in the histopathology of colon tissue was accompanied by an increase in RNA level of TJ proteins, resulting in the improvement of liver histopathology in an HFD/CCL_4_-induced mouse model.

In a previous study conducted by our team of researchers, it was found that *A. muciniphila* and its EVs reduced the adipose size, inhibited inflammation, and increased mucosal thickness and crypt depth in the colon tissue of HFD-induced obese mice^37^. Grander et al. also found that three doses of live *A. muciniphila* could reduce liver damage and restore the intestinal barrier integrity in mice with alcohol-induced liver injury^38^. Moreover, Wu et al. reported that treatment with live *A. muciniphila* for 14 days led to the reduced infiltration of neutrophils and macrophages in an immune-mediated liver injury mouse model^9^.

It has been shown that in methionine-choline deficient diet (MCD) mice, steatosis, inflammation, and fibrosis reduce, and the histological futures of the liver tissue improve via induction of insulin-like growth factor (IGF)^39^. Another study showed that use of *igf* could inactivate the effect of IL-6 and the inflammatory response in the HepG2 cell line^40^. The results of the present study showed that the mRNA level of *igf* gene decreased in the PBS group as compared to the healthy control group, while gavage with live and pasteurized *A. muciniphila* and its EVs remarkably restored it in the liver tissue. Interestingly, EVs showed a much better effect on inducing the expression of *igf* gene compared to the other study groups. Therefore, the *igf* gene is necessary for insulin sensitivity, with an important role in hormonal and metabolic pathways involved in liver protection^41^. *IGF* deficiency can cause chronic liver disease (CLD) and primary liver cancer; therefore, an understanding of this condition can provide new insights into the control of liver disease^42,43^.

Inflammation can be initiated by different pathogen-associated molecular patterns (PAMPs) and damage-associated molecular patterns (DAMPs) through the expression of toll-like receptors (TLRs) in the liver^44^. TLRs are one of the most important members of the pattern recognition receptor (PRR) family, expressed in most types of liver cells. *tlr*-*2, tlr*-*4*, *tlr*-*5*, and *tlr*-*9* genes have been studied more extensively than other types of TLRs in liver diseases^45^. The *tlr-5* gene activates NF-κB receptors and has been shown to play a major role in many pro-inflammatory responses^46^. Recent studies have reported that the liver is the organ that primarily responds to flagellins by activating the TLR5-MyD88 signaling pathway^47,48^. The role of *tlr-9* gene in liver disease has been also investigated, and it has been found that *tlr-9*-defective mice are resistant to alcoholic fatty liver disease^49^. Overall, activation of *tlr*-*9* gene can lead to liver damage, associated with the increased transmission of bacterial compounds to systemic circulation, a phenomenon observed in many liver diseases^50^.

We previously reported that heat-killed *A. muciniphila* could decrease *tlr*-*2* and *tlr*-*4* mRNA levels in the LPS-activated LX-2 cell lines and induce HSC regression^25^. In our recent project, we also found that administration of live and pasteurized *A. muciniphila* and its EVs reduced mRNA levels of different fibrosis markers e.g. *α-sma, timp, pdgf, tgf-β* and *tlr-2* and *tlr-4* in a similar mouse model^51^. In another study on normal-diet mice also we observed that live and pasteurized *A. muciniphila* reduced the mRNA level of *tlr-4, tnf-α, tgf-β* and increased *il-10, ppar-α* and *ppar-γ* in liver tissue suggesting these bacteria can be considered as new medical supplement to maintain health state and prevent diseases in normal mice^52^. Therefore, in current study we focused on *tlr5* and *tlr9* and our results showed that in both quiescent and LPS-activated LX-2 cells, neither live nor pasteurized *A. muciniphila* altered the mRNA expression of *tlr-5* gene, while EVs could ameliorate the expression of *tlr-5* gene at a concentration of 50 µg. Treatments could not affect the mRNA level of *tlr-9* gene in quiescent LX-2 cells, while in LPS-activated LX-2 cells, all treatments showed inhibitory effects on *tlr-9* genes; the effect of EVs was dose-dependent. In the HFD/CCL_4_ mouse model, the use of all three supplementations of *A. muciniphila* decreased the expression of *tlr*-*5* and *tlr*-*9* genes in the liver tissue.

It has been demonstrated that *tlr-9* knockout mice with a choline-deficient L-amino acid-defined (CDAA) diet are protected against lipid accumulation, steatohepatitis, and liver fibrosis through suppressing *IL-1β* production^53^. A recent study by Zhou et al. on *tlr-5* knockout mice showed that *tlr-5* signaling modulates CCl_4_-induced liver fibrosis by inducing interferon β (IFN-β) expression and regulating *IL*-*1β* receptor antagonists (IL-1RA) in mice^54^. In contrast, in another study on *tlr-5*-deficient mice, the role of *tlr-5* in CCl4-induced liver fibrosis was investigated. The results showed that *tlr-5* was directly involved in the progression of fibrosis by activating the NF-κB and MAPK signaling pathways. Also, in *tlr-5* knockout mice, CCl_4_-induced hepatic fibrosis was reduced by inhibiting smooth muscle alpha-actin (α-SMA) and collagen expression. Therefore, *tlr*-*5* is involved in the progression of fibrosis and may directly or indirectly enhance the development and formation of liver fibrosis by activating HSCs through interactions with other TLR families^55^.

Overall, the findings regarding the exact role of *tlr*-*5* in the prevention or induction of liver fibrosis are contradictory, considering the limitations or different models used to induce liver damage. According to the results of the present study and the proven beneficial effects of *A. muciniphila* on the function of the intestinal-epithelial barrier, it can be concluded that the use of *A. muciniphila* and its derivatives has favorable effects on the liver tissue by improving the intestinal barrier integrity and decreasing the expression of *tlr*-*5* and *tlr*-*9* genes.

The TLR-dependent HSC stimulation activates Kupffer cells and NF-κB pathways, followed by the production of TGF-β and various pro-inflammatory cytokines, such as IL-6, IL-1β, TNF-α, and IL-8^56^. IL-6, which can be produced by the liver, is an important determinant of acute-phase response proteins, such as C-reactive proteins (CRP), serum amyloid A (SAA), hepcidin, and other factors originating from the liver tissue^57^. It is clear that IL-6 directly or indirectly stimulates the liver cells and is involved in the development of chronic hepatitis^58^. In contrast, IL-10 is a type II cytokine with anti-inflammatory effects, which plays an essential role in preventing inflammatory and autoimmune damages^59^. Besides, neutralization of IL-10 using antibodies exacerbates hepatic steatosis and insulin resistance in mice^60^. Hepatic and extra-hepatic sources of IL-1β, upregulated by activated macrophages, contribute to metabolic liver inflammation steatosis and fibrosis^61,62^.

The results of the present study showed that daily gavage with *A. muciniphila* and its derivatives not only decreased *il*-*6* gene expression and increased *il*-*10* expression in the liver tissue, but also decreased the tissue levels of these inflammatory and anti-inflammatory cytokines in the liver and adipose tissues of HFD/CCL_4_ mice. The mRNA level of *il*-*1β* gene also remarkably decreased in the liver and adipose tissues of mice, gavaged with live and pasteurized *A. muciniphila* and its EVs. As mentioned earlier, this inhibition of *il*-*1β* was accompanied by a decreased *tlr*-*9* mRNA level in the liver tissue.

Moreover, the results of an in vivo study showed the protective effects of a combination of *Lactobacillus* (*L. paracasei* and *L. casei*) and *Weissella* species on thioacetamide-induced liver injury (TAA). They also found that TAA-treated mice receiving probiotics had significantly less liver damage, which was associated with the reduced liver protein content of TNF-α^63^. Moreover, in vitro and in vivo data from a study by Ashrafian et al. showed that both *A. muciniphila* and its EVs had stimulatory effects on the expression of *il*-10 as an anti-inflammatory cytokine. Besides, EVs induced the production of fewer pro-inflammatory cytokines, such as IL-6, IL-8, and IFN-γ as compared to the live bacterium^37^.

A recent study by Huck et al*.* showed that *A. muciniphila* could counteract the destructive inflammatory effects of *Porphyromonas gingivalis* in lean and obese animals by inducing the expression of *il*-10 and reducing pro-inflammatory cytokines^64^. Also, a study by Kang et al. showed that pretreatment with *A. muciniphila*-derived EVs reduced the production of IL-6 from clone epithelial cells (CT-26), treated with *Escherichia coli* EVs^62^. Besides, studies have shown that IL1-β plays a key role in adipose and liver inflammation, which is in line with our observations^65,66^. Therefore, induction of IL-10 as an anti-inflammatory cytokine and decreased production of IL-6, TNF-α, and *il1*-β as pro-inflammatory cytokines by *A. muciniphila* and its EVs indicate the anti-inflammatory properties of both forms of this bacterium on the liver tissue. In our previous study we also observed that EVs more efficiently reduced the serum level of IL-6, TNF-α and induced IL-10 than other groups^51^. This anti-inflammatory effect of EVs can be attributed to the presence of certain compounds in EVs, which regulate immune homeostasis in the intestine, adipose tissue, liver, and other body organs.

PPAR-α and PPAR-γ are predominantly involved in reducing inflammatory responses, with protective effects against acute liver injury^67^. PPAR-γ plays an anti-fibrotic role by inhibiting downstream TGF-β signaling transduction in the liver pro-fibrotic pathways^68^. The PPAR-α pathway has also been shown to be involved in hepatitis and fibrosis and inhibit the activity of inflammatory transcription factors, including NF-κB and activating protein 1 (AP-1)^69^. Our results showed that the use of *A. muciniphila* and its EVs increased the expression of both *ppar*-α and *ppar*-γ genes in the LPS-activated LX-2 cell line. In the HFD/CCL_4_ mouse model, a decrease in the expression of these two genes was observed in the PBS group, while in the other treatment groups, a significant increase in the mRNA level of *ppar*-α and *ppar*-γ genes was observed in both adipose and liver tissues.

In this regard, Choi et al. showed that PPAR-γ agonists could prevent TGF-β-induced fibrogenesis. They found that PPAR-γ is a new target in the treatment of liver fibrosis and inflammation^70^. A recent study also showed that administration of *Lactobacillus amylovorus* for 12 weeks reduced the adipocyte and plasma cholesterol levels and also increased the expression of hepatic and adipose *PPAR*-α^71^. Consistent with this study, our previous study also showed that *A. muciniphila* and its EVs increased the expression of *ppar*-α and *ppar*-γ in the adipose tissue of HFD-fed mice and improved fatty acid oxidation and energy metabolism^37^. We have also previously reported that the mRNA level of *ppar*-γ gene was significantly reduced in activated HSCs and interestingly heat-killed *A. muciniphila* could prevent HSCs activation through increasing *ppar*-γ mRNA level in LPS-activated LX-2 cells^25^. Therefore, since PPAR activation could prevent fatty acid accumulation and regulate inflammatory responses^72^, it can be concluded that *A. muciniphila* and its derivatives might have anti-inflammatory effects on mice with HFD/CCL_4_-induced liver fibrosis through induction of PPAR gene expression.

In conclusion, the present results showed that oral administration of *A. muciniphila* and its derivatives for four weeks could enhance anti-inflammatory responses of the colon, adipose, and liver tissues and subsequently prevent liver injury in HFD/CCL_4_ mice. However, we acknowledge several limitations in this study. The main limitation of this study was that we did not determine the content of EVs isolated from *A. muciniphila*; therefore, we could not explain the exact beneficial mechanism of these bacterial derivatives in the liver function and intestinal hemostasis. Further studies are needed to confirm the effects of *A. muciniphila* and its derivatives on the prevention of liver injury in humans and explore their potential risks and adverse effects.
